# Trust-based service innovation of municipal home care: a longitudinal mixed methods study

**DOI:** 10.1186/s12913-022-08651-6

**Published:** 2022-10-15

**Authors:** Tom Eide, Monika K. Gullslett, Hilde Eide, Janne H. Dugstad, Brendan McCormack, Etty R. Nilsen

**Affiliations:** 1 Centre for Health and Technology, Faculty of Health and Social Sciences, University of South-Eastern Norway, Drammen, Norway; 2 Faculty of Medicine and Health, The University of Sydney, Camperdown, Australia; 3 Faculty of Health and Social Sciences, University of South-Eastern Norway, Drammen, Norway

**Keywords:** Homecare, Trust-based management, Service innovation, Complexity, Person-centredness, Motivation, Purchaser-provider split, Leadership anxiety

## Abstract

**Background:**

In Scandinavia, various public reforms are initiated to enhance trust in the healthcare services and the public sector in general. This study explores experiences from a two-step service innovation project in municipal home care in Norway, coined as the Trust Model (TM), aiming at developing an alternative to the purchaser-provider split (PPS) and enhancing employee motivation, user satisfaction, and citizen trust. The PPS has been the prevalent model in Norway since the 1990s. There is little empirical research on trust-based alternatives to the PPS in healthcare. The overall objectives of this study were to explore facilitators and barriers to trust-based service innovation of municipal homecare and to develop a framework for how to support the implementation of the TM.

**Methods:**

The TM elements were developed through a comprehensive participatory process, resulting in the decision to organize the home care service in small, self-managed and multidisciplinary teams, and trusting the teams with full responsibility for care decisions and delivery within a limited area. Through a longitudinal mixed methods case study design a) patients’ expressed values and b) factors facilitating or preventing the service innovation process were explored through two iterations. The first included three city districts, three teams and 80 patients. The second included four districts, eight teams and 160 patients.

**Results:**

The patient survey showed patients valued and trusted the service. The team member survey showed increased motivation for work aligned with TM principles. Both quantitative and qualitative methods revealed a series of facilitators and barriers to the innovation process on different organizational levels (teams, team leaders, system). The key message arising from the two iterations is to keep patients’ values in the centre and recognize the multilevelled organizational complexity of successful trust-based innovation in homecare. Synthesizing the results, a framework for how to support trust-based service innovation was constructed.

**Conclusions:**

Trust-based innovation of municipal homecare is feasible. The proposed framework may serve as a tool when planning trust-based innovation, and as a checklist for implementation and improvement strategies. Further research is needed to explore the validity of the framework and its replicability in other areas of healthcare.

## Background

### New Public Management (NPM) and the challenge of trust

There is a reported decline in trust in Western healthcare systems [1–3]. This development seems to go parallel with the decline in service provider autonomy in healthcare organizations, as a result of the dominant role of NPM thinking over the last 20–30 years [4, 5]. In Scandinavia, various trust reforms are initiated on national and municipal levels to restore trust in professionals and the public sector in general [6–8]. The backdrop of organization studies during the 1990s indicated that trust in team members is key to efficient and effective performance [9–11].

The purchaser-provider split (PPS) model has been the prevalent one in Norwegian and most European health systems since the introduction of NPM in the 1990s. The PPS is a service delivery model in which third-party payers are kept organizationally separate from service providers [12]. In the Nordic countries, the public sector traditionally fills the role of both purchaser and provider, financed by global budgets and without specification of expected volume or quality. Since the early 1990s the need to introduce incentives to manage scarce resources more efficiently gradually emerged as a policy issue [13]. In Scandinavia, Sweden was the main innovator, various purchaser–provider split arrangements were introduced in the late 1980s, in an effort to stimulate market-style competition, enhance efficiency in the public sector and widen patient choice [14]. In Norway, a purchaser-provider separation was first introduced for nursing and care services in the early 1990s [15]. The need for enhanced efficiency, quality and equity was the main rationale when PPS models were introduced [16, 17]. However, the recommendations to do so were ambiguous. The Norwegian Government’s green paper introducing the PPS [18] pointed to the possible risks of a too strict separation between the purchaser and provider; increased distance between the decision-maker and the service user; unwanted growth of bureaucracy, and delay of decision procedures, which especially might disadvantage persons with complex care needs. The research literature on the implication of PPS for the quality of homecare services is limited.

### The Trust Model innovation project

This study explores facilitators and barriers to trust-based service innovation in the case of a two-step service innovation project in municipal homecare, coined as the Trust model (TM). By “service innovation” we mean the process of developing a way of organizing and providing healthcare that is perceived as new by affected parties, like patients and service providers. The aim of this innovation project was to develop a trust-based and person-centred alternative to the PPS-model, to increase patient satisfaction, employee motivation and citizens’ trust in the municipal homecare services. The term “person-centred” here applies to healthcare practice, and the political intention of increasing user participation and shared decision making concerning care delivery [19, 20]. The person-centred approach [21] of focusing patient values and priorities (“What matters to you?”) was also anchored both in the participatory process and in national policy documents [22, 23].

The TM innovation project was politically initiated by the City Council of Oslo, originally inspired by the trust reform of the municipality of Copenhagen, Denmark [7] and the Dutch private *Buurtzorg* model, providing homecare through self-managed teams of nurses [24]. The assumptions implicit in the political decision of developing the TM were that trust-based organizational change would increase team members’ motivation, patient satisfaction and citizens’ confidence in municipal healthcare.

Four of the 15 city districts of Oslo took part in the project, headed by a steering group consisting of senior managers and team members’ union representatives of the four districts. A key learning from the Copenhagen project was that organizational trust does not follow automatically from strategic top-down decisions [25]. Hence, a comprehensive participatory bottom-up process was put in place across the four districts during 2016 and 2017 to develop proposals for a trust model in homecare.

The steering group decided on a proposal consisting of three core elements: a) organizing in small, self-managed and multidisciplinary teams (about 12 persons), b) trusting the teams with full responsibility for all persons in need of homecare in a limited geographical area (maximum about 150 patients), and c) taking the question “What matters to you?” as a starting point for patient involvement and care decisions.

Whilst aligned with the Buurtzorg model of self-managed teams, the TM deviates from this model both by its multidisciplinarity (vs unidisciplinary/nursing), the scope of team responsibility (about 150 vs about 50 patients per team), and by being an integrated part of a public, municipal healthcare system responsible for all patients in the area (vs private service delivery responsible for a selection of patients).

We here apply the term “patient” to all persons receiving homecare, i.e., service users or recipients. The term “multidisciplinary” here implies that the basic staffing of each team should include both the professions of nursing, physiotherapy and occupational therapy as well as homecare workers and administrative officers [26]. By “team members” we here mean the multiprofessional staff, including service providers and administrators.

Municipality policy expectation from the development and implementation of the TM were to a) increase patient safety, satisfaction and participation, b) increase team members motivation and work satisfaction, and c) increase service flexibility, efficiency and quality [19, 20, 27].

When the concept of the TM was developed and the first two innovation iterations took place (2016–18), there was little empirical research to learn from. Even though trust in public organizations has been a hot topic of political and professional debate during the last decade, there is still a research gap concerning facilitators and barriers to support trust-based service innovation in health and social care. This case study of service innovation in municipal homecare aims at contributing to filling this gap.

### The complexity of the concept of trust and theoretical assumptions guiding the study

The concept of trust implicit in the terms “trust model” and “trust-based” is complex. On the macro level of the healthcare system, it applies to a political intention of improving the service quality and increasing citizen trust in the municipal services. On the meso level of the homecare organization where the service innovation project was situated, it applies to the political intention of increasing healthcare professionals' responsibility, authority, and area of discretionary powers. On the micro level of homecare practice, it applies to the interpersonal relationships between the patient and healthcare professionals, and the increasing emphasis on person-centredness, involvement and autonomy. Aware of these three levels of trust, the theoretical backdrop of the study is comprised of three complementary theories of trust, respectively Luhmann's systems theory, conceptualizing trust as a means to reduce social complexity [28], Mayer, Davis & Schoorman's model of organizational trust, suggesting ability, benevolence and integrity as the key preconditions of perceived trustworthiness [11, 29], and Løgstrup's relational ethics, suggesting a natural human condition of basic trust to be the ontological root of moral responsibility [30].

### Objectives and research questions

The overall objectives of this study were to explore facilitators and barriers to trust-based service innovation of municipal homecare and to develop a framework for how to support the implementation of trust on different organizational levels. The following research questions were asked:

1. What do the patients value or need, i.e., what do the patients report as most important to trust and be satisfied with the service?
2. What are team members prerequisites, i.e., what do staff require to meet patient needs?
3. What are the team leader prerequisites, i.e., what do leaders require to support teams to meet patient needs?
4. What are the key system prerequisites, i.e., what should the municipal healthcare organization put in place to enable the model (TM) to thrive (as an alternative to the PPS)?

## Methods

### Design

The study was exploratory, and draws on a longitudinal single-embedded and convergent mixed methods case study design [31, 32], with elements of action research in the sense of a change process accompanied by systematic inquiry, reflection, learning and new action [33]. Both qualitative and quantitative methods were used to explore background and context of the project and experiences and perspectives of affected parties on different levels of the innovation project, i.e., those of the a) patients, b) team members and c) managers (team leaders, unit leaders, project leaders, etc.). Table 1 gives an overview of the data collection, methods, participants in the specific activities and the timeline through the project.

**Table 1:** Data collection methods, informants and timeline

***Methods & informants***	***Timeline***													
	***First iteration***						***Second iteration***						***Evaluation***	
	***2016 *Aug.**	***2016 Sept.***	***2016 Oct.***	***2016 Nov.***	***2016 Dec.***	***2017 Jan.***	***2017 Feb.***	***2017 March***	***2017 April***	***2017 Oct.***	***2017 Nov.***	***2017 Dec.***	***2018 Jan.***	***2018 April***
***Questionnaire patients***				***x***				***x***			***x***		***x***	
***Questionnaire team members – part 1 and 2***				***x***							***x***			
***Questionnaire team members – part 3***						***x***							***x***	
***Questionnaire team members – part 4***													***x***	
***Workshop IGP-process and interviews with all teams***										***x***	***x***	***x***		
***Participative observation at workshops***^a^		***x***				***x***		***x***						
***Observation team meetings***				***x***	***x***	***x***	***x***							
***Observation project group***	***x***	***x***						***x***	***x***					
***Participative observation team leader group***^a^						***x***			***x***					
***Participative observation steering group***^a^			***x***					***x***						
***Participative observation in diverse meetings***			***x***	***x***	***x***	***x***	***x***	***x***	***x***		***x***	***x***	***x***	
***Conversations and meetings with main project leader***	***x***	***x***	***x***	***x***	***x***	***x***	***x***	***x***	***x***		***x***	***x***	***x***	
***In depth leader interviews***			***x***									***x***		
***Document analysis***	***x***	***x***	***x***					***x***	***x***				***x***	
***Participative observation evaluation/validation process***														***x***

^a^Including presentation of findings

### Participants

The data collection was undertaken during 2016–2018, including two iterations (TM pilots) and an evaluation process (see Table 1). The first iteration (autumn 2016) included three city districts, six multidisciplinary self-managed teams and 80 patients. The second (2017) included one more district, four districts in all, eight teams and 160 patients.

Table 2 provides and overview of the characteristics of participating homecare patients in the four city districts. Most of these patients were above 80 years old, about a third in their seventies and a few under 67 years of age. About 70% of the patients were women (see Table 2).

**Table 2:** Characteristics of participating home care patients in the four city districts

		**T1—Nov. 2016** ***N* = 79**	**T2—March 2017** ***N* = 73**	**T3 – Nov. 2017** ***N* = 210**	**T4 – Jan. 2018** ***N* = 160**
District N (%)	1	13 (9.8)	16 (21.9)	34 (16.2)	43 (26.9)
	2	27 (20.5)	25 (34.2)	64 (30.5)	23 (14.4)
	3	39 (29.5)	33 (45.2)	68 (32.4)	59 (36.9)
	4	53 (40.2)	0	44 (21.0)	35 (21.9)
Gender N (%)	Male	40 (30.5)	19 (26)	59 (28.1)	52 (32.5)
	Female	91 (69.5)	52 (74)	151 (71.9)	108 (67.5)
Age N (%)	18–49	1 (0.8)	3 (4.1)	3 (1.4)	2 (1.2)
	50–67	18 (13.6)	11 (15.1)	16 (7.7)	16 (9.9)
	68–80	40 (30.3)	15 (20.5)	49 (23.4)	36 (22.4)
	81–100	73 (55.3)	41 (56.2)	141 (67.5)	107 (66.5)

The participating team members represented a broad spectrum of positions, competences, and educational backgrounds, most of them RNs and health workers, but also executive officers, physiotherapists, occupational therapists, practical assistance providers and others (see Table 3). The districts had varied socio-cultural profiles and between 27,000 and 50,000 inhabitants. Within the context of municipal health services of the City of Oslo, all city districts were autonomous concerning management of their homecare services.

**Table 3:** Overview of characteristics of participating team members in the four city districts

		**Nov. 2016** ***N* = 153**	**Jan. 2017** ***N* = 29**	**Nov. 2017** ***N* = 76**	**Jan. 2018** ***N* = 60**
District N (%)	1	47 (31)	8	17 (23)	8 (13)
	2	32 (21)	11	14 (18)	16 (27)
	3	32 (21)	10	22 (29)	21 (35)
	4	42 (27)	0	23 (30)	15 (25)
Gender N (%)	male	27 (18)		17 (22)	12 (20)
	female	126 (82)		59 (78)	47 (80)
Age	years (median min–max)	43 (23–63)	42 (25–60)	44 (19–63)	45 (18–60)
Profession/ position N (%)	Registered nurse	45 (30)	14 (48)	22 (29)	18 (30)
	Nurse/health worker	41 (27)	6 (22)	20 (26)	25 (42)
	Executive officer	18 (12)		5 (7)	4 (7)
	Physiotherapist	5 (3)	3 (10)	4 (5)	2 (3)
	Occupational therapist	6 (4)	1 (3)	5 (7)	2 (3)
	Part time employee	15 (10)			2 (3)
	Practical assistance provider	11 (7)	1 (3)	2 (2)	2 (3)
	Other		4 (14)	10 (13)	
	Missing	12 (8)		9 (12)	5 (8)

All four districts participated in the first survey, but one of the districts (district 4) withdrew from participation in the first iteration (therefore 0 participants March 2017, Table 3).

Table 3 provides an overview of the participants in the team member survey in the two iterations. In the first iteration, 200 questionnaires were distributed to team members in the homecare services, 50 per district (response 76%). At the beginning and end of the second iteration questionnaires were distributed to the 105 participating team members (response 73%). The respondents represented a broad spectrum of positions, competences, and educational backgrounds.

### Quantitative methods—questionnaires

#### The patient survey

To explore the patients’ values regarding trust and satisfaction with the services, we designed a survey with both closed and open questions. The “Patients´ trust in the services” survey consists of six questions designed specifically for this study with scale response from 1–5 (1 = “to a very small extent” to 5 = “to a very large extent”). In addition, patients answered three open questions about what it was about the service they were happy with, what they were not so happy with, and demographic questions. The survey was completed anonymously, and patients needing help were assisted by a research assistant, not a team member.

#### The team member survey

To explore the team members’ perspectives and experiences of factors facilitating or preventing the transition to trust-based, self-managed teams and new procedures based on what the patient considers important to him or her, a survey with 4 parts was compiled. Part 1 and part 2 were developed specifically for this study and administered at the start of each iteration (respectively Nov. 2016 and Nov. 2017).

Part 1 “*Team members trust and self-determination*” were measured with five questions about the degree of trust and self-determination at work. The response scale goes from 1 = “to a very small extent” to 5 = “to a very large extent”. Cronbach’s alpha for the total sum-score of the scale was 0.90 in both iterations, indicating excellent internal consistency.

Part 2 “*Team members motivation at work”* was measured with four questions with a response scale from 1–5 (1 = “to a very small extent” to 5 = “to a very large extent”). Part 3 and part 4 are validated questionnaires and administered at the end of each iteration (respectively Jan. 2017 and Jan. 2018).

Part 3 *Person-centred Practice Inventory - Staff (PCPI-S****)*** [34, 35] consists of 59 questions concerning person-centredness and person-centred culture. The PCPI is divided into three main areas: (1) prerequisites for person-centredness, (2) care processes and (3) care environment. The answer options range from 1 = “totally disagree” to 5 = “completely agree”.

Part 4 “*Measuring Instrument for Determinants of Innovations*” (MIDI) questionnaire [36, 37] was a mapping of the team members’ evaluation of the prerequisites for implementation. This instrument captures four different categories or groups of determinants of innovation, those associated with (a) the socio-political context related to legal regulations (1 determinant), (b) the innovation itself (here: the TM; 7 determinants), (c) the users of the innovation, here the team members, and the degree to which the team members believe the innovation is relevant to his or her patients (11 determinants), and (d) the organization where the TM is implemented (10 determinants). The scale range is from 1–5 (1 = totally disagree, 5 = totally agree).

### Qualitative methods

Multiple qualitative methods were used to explore the background and context of the innovation project and participating stakeholder experiences and perspectives of the processes used.

#### Document analysis

To explore background and context of the TM a series of documents were analysed, including white papers, green papers, municipal policy papers, minutes of steering group meetings and process documents concerning the participatory development process of the TM.

#### Workshop process interviews

To explore team members’ experiences and perspectives, process interviews across the teams were designed in IGP format (individual-group-plenary). Three IGP process workshops were conducted with about half of the team members present each time, each workshop with a specific trust-related topic. The process interviews were organized in three steps. First (individual) each participant completed a questionnaire developed for the specific workshop, including open questions about what worked well and less well. Second (group) team members were sharing their answers and reflections with each other. Third (plenary) each team wrote key points on a large piece of paper, put it up on the wall and presented them to the other teams for them to respond to, including their main concerns, ideas for improvements and a narrative illustrating their core experience so far. Data were collected through the questionnaires and observational notes. In addition, to enable collection of rich process data, IGP process was chosen for action research reasons, in order to stimulate reflection and organizational learning, trust building and innovation across teams, which seems to be an outcome of IGP processes in inter-firm networks [38, 39].

#### Participatory observation

To explore team members’ and managers’ experiences and perspectives, participatory observation was conducted in team meetings, managers’ meetings (steering group, project leader group, etc.) and other meetings where members of the research group presented preliminary results to managers, team members and other healthcare personnel.

#### Leadership interviews

To explore managerial-level experiences and perspectives further, 10 individual in depth interviews were conducted at the end of the second iteration (December 2017) with leaders at different levels (the leader of the steering group, the main project leader, the team leaders, the local project managers, a unit leader and a team members’ union representative). The interview guide was developed from the preliminary analyses of the observational data, the patient and team member surveys, and the IGP process interviews. The interviews were conducted as open, exploratory conversations, using the interview guide as a checklist.

### Data analysis

In accordance with a convergent mixed-methods design [32], the quantitative and qualitative data were first analysed separately, and then the data were combined to propose an integrated model of how to support successful implementation of trust-based homecare.

#### Quantitative analysis

The questionnaire data were analysed with SPSS [40]. Descriptive statistics were used to describe the samples. ANOVA-analysis for independent groups were used to determine group differences and possible change over time. Internal consistency of the scales and subscales were assessed by Cronbach’s coefficient α [41]. For determining if a MIDI-determinant could be regarded a facilitator or a barrier; MIDI items to which ≥ 20% of participants responded ‘totally disagree/disagree’ were regarded as barriers and items to which ≥ 80% of participants responded ‘agree/totally agree’ as facilitators [42]. Cohens D was calculated to determine the degree of change [43].

#### Qualitative analysis

The qualitative data collected through observation, questionnaires and group and individual interviews were analysed according to phenomenological-hermeneutical procedures [44], trying to identify and categorize essential meanings, i.e. the informants’ lived experience of facilitators and barriers to trust-based innovation. The data were read (and the taped interviews listened to) several times by researchers with different scholarly backgrounds (organization studies, sociology, psychology, ethics, and health sciences), moving from a first naïve reading through several rounds of structural analysis towards a more comprehensive understanding of the whole.

### Combining the quantitative and qualitative data to construct a framework

During both iterations preliminary qualitative and quantitative research findings were presented to and discussed with the homecare teams, the project leader group and the main project leader to establish the trustworthiness of the analysis. At the end of both iterations, findings were presented to and discussed with the steering group and published in evaluation report format, including recommendations for further improvement of the trust model [45, 46]. The results of these reflective discussions were included in the data.

To construct a framework for trust-based homecare innovation and meet threats to trustworthiness, we repeatedly went back and forth between the different qualitative and quantitative data sources in a cross-disciplinary four steps interpretation process according to the research questions and their context of organizational level. The final step of the analysis was to adjust and formulate the key findings in a phenomenological way, i.e. in an everyday language as close to lived experience as possible [44].

## Results

**Fig. 1:**
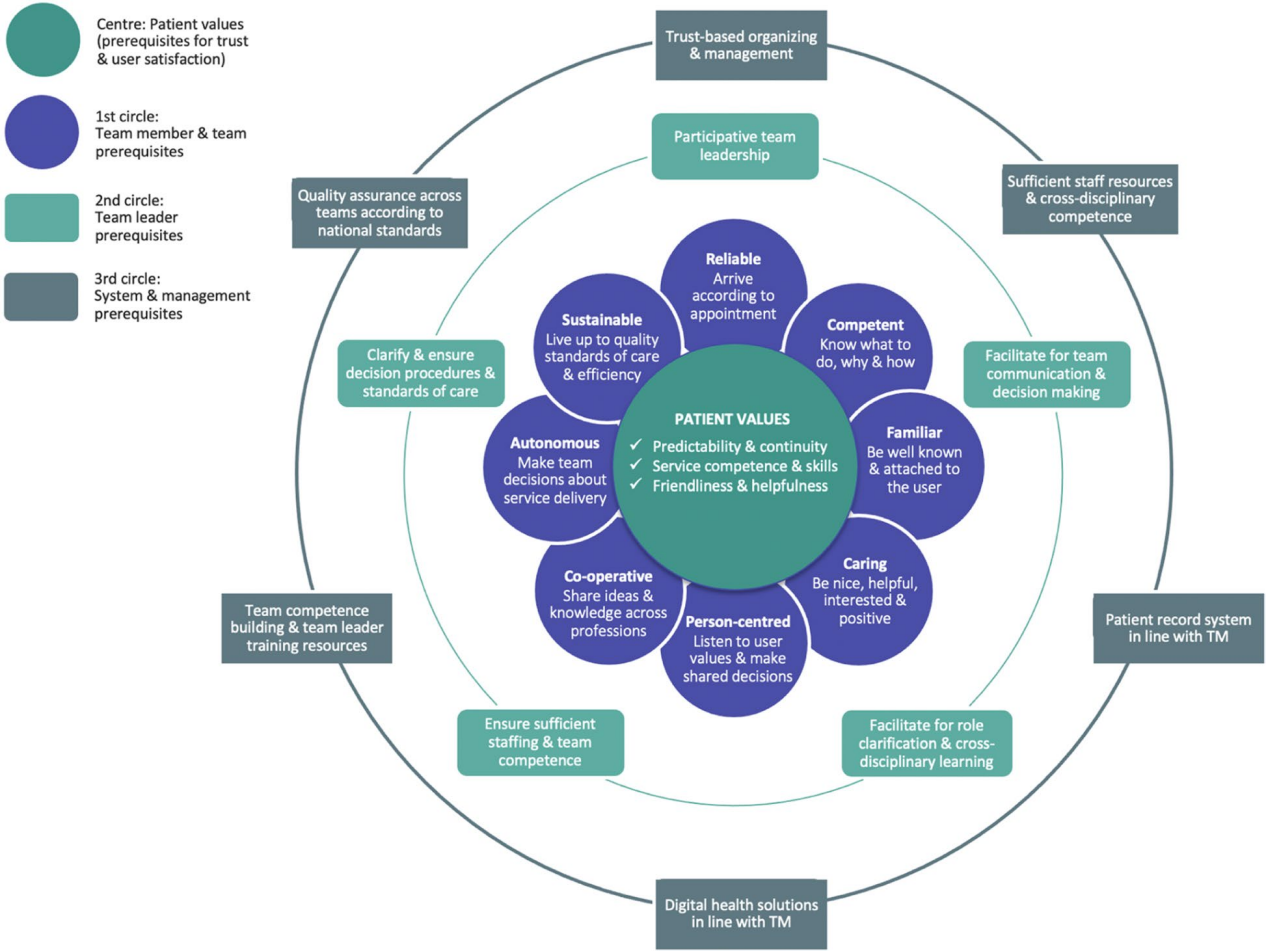
An integrative framework for trust-based service innovation of homecare

We present the results from the different data sources organized according to the research questions; patients, team members and leaders, and the different methods; surveys, interviews (individual and group) and observations. A synthesis of the findings from the multitude of data sources is presented in the form of a framework for trust-based innovation of homecare (Fig. 1).

### Patients’ values

We present the results of the patient surveys in the two iterations together to illuminate the development.

#### The participating patients’ trust in the services (Table 4)

**Table 4:** The patient survey: Patients´ trust in the services

**Question** **To which degree..**	**Nov. 2016** ***N*** ** = 79**	**Jan. 2017** ^**b**^ ***N*** ** = 73**	**Nov. 2017** ***N*** ** = 207**	**Jan. 2018** ***N*** ** = 162**
1. do you feel confident that you will receive the help you need from the municipality?	3.63 (0.9) 1-5^a^	3.95 (0.8) 2-5^c^	3.66 (0.91)	3.82 (0.82)
2. do you participate in the decision making concerning the help you receive?	3.15 (1.1) 1–5	3.37 (1.2) 1–5	3.43 (1.01)	3.51 (0.91)
3. do you feel that your wishes and objectives are taken into account when decisions about services are made?	3.35 (1.1) 1–5	3.59 (0.9) 1–5	3.52 (0.96)	3.54 (0.91)
4. does the home care service adjust to your needs when your needs change?	3.37 (1.1) 1–5	3.70 (1.0) 1-5^c^	3.52 (0.93)	3.58 (0,82)
5. do you receive help with what it is important to you to get help with?	3.72 (0.9) 1–5	3.82 (1.0) 1–5	3.69 (0,90)	3.83 (0.90)
6. How satisfied are you with the home care services you receive from the municipality?	3.66 (0.9) 2–5	3.89 (0.9) 1.5	3.76 (0.79)	3.85 (0.82)
**Mean sum score** ^e^	**3.48 (0.8)**	**3.75 (0.8)** ^d^	**3.61 (0.8)**	**3.68 (0.7)**

^a^Mean (SD) min–max, scale range 1 = “to a very small extent” to 5 = “to a very large extent”

^b^At T2 district 4 does not participate

^c^*p* < 0.05 for difference mean value single questions

^d^*P* = 0.044, T-test for independent groups

^e^Cronbachs alfa sum score was 0.90 in both iterations

Patients were largely confident that they would get assistance when needed with what was most important to them. They were also largely satisfied with the homecare service. An average summary of the five questions can be viewed as a total assessment of the patients’ experienced quality of the services, including confidence and trust. The answers are close to the category “largely agree”. During the first iteration there was a statistically significant improvement in patient satisfaction. When the second iteration started, patient satisfaction was on the same level as at the end of the first and remained stable at that level.

The patients answered open questions about what they liked the most and least. What was most important to the patients can be grouped under three headings, a) *predictability and continuity,* i.e. that the professionals keep appointments, “not coming late”, and that there are not “too many unknown people coming”, b) *competence and skills*, i.e. that they “know what to do”, “do a good job” and bring the correct medication or necessary devices with them, and c) *friendliness and helpfulness*, i.e. be “caring”, “nice, positive and friendly”, show interest and have time to listen.

### Team members’ perspectives on the service and innovation process

#### Trust and self-determination (Table 5)

**Table 5:** The team member survey: *Part 1 Team members´ trust and self-determination*

**To what degree…**	**Nov. 2016** ***N*** ** = 153** **Mean (SD) min–max**	**Nov. 2017** ***N*** ** = 76** **Mean (SD) min–max**
1. do you feel you have self-determination at work?	3.34 (0.8) 1–5^#^	3.30 (0.8) 1–5
2. are you at present trusted as a professional?	3.77 (0.8) 2–5	3.66 (0.8) 1–5
3. do you want self-determination at work?	3.89 (0.7) 1–5	3.99 (0.6) 3–5
4. do you feel that your own and your colleagues’ proposals for service improvements are well received?	3.2 (0.86) 1–5	3.18 (0.8) 1-5^a^
5. do you feel that you are free to find good solutions together with the service user?	3.35 (0.9) 1–5	3.45 (0.7) 1–5

^#^Scale range min–max: 1 = “to a very small extent” to 5 = “to a very large extent”

^a^Statistically significant difference between the districts, *P* = 0.000

The team members wanted a high degree of self-determination at work. They experienced a moderate degree of professional trust. The entire response scale (1–5) was used, indicating large variations on an individual level (See Table 5).

At the start of the first iteration, 83% of the team members answered yes and 17% no to the question whether they believed that the TM would give better services for the patients than the PPS model. At the beginning of the second iteration the score was even higher, 96% yes and 4% no.

#### Motivation at work (Table 6)

**Table 6:** The team member survey: *Part 2 Team members´ motivation at work*

**To what degree…**	**Nov. 2016** **Mean (SD)**	**Nov. 2017** **Mean (SD)**	**«Effect»** **Cohens D**
1. has working according to the TM influenced your motivation in a positive way?	4.19 (0.8)	3.74 (0.9)^a^	- 0.53
2. has working according to the TM influenced your motivation in a negative way?	2.26 (0.9)	2.12 (1.2)^a^	-
3. has working according to the TM influenced other team members’ motivation in a positive way?	3.96 (0.6)	3.60 (0.9)^a^	- 0.47
4. has working according to the TM influenced other team members’ motivation in a negative way?	2.41 (0.8)	2.39 (1.2)^a^	-

^a^Statistically significant difference between the districts

The majority of team members reported that working with the TM positively influenced their work. The same tendency was found when they were asked about the motivation of the team as a group. From the end of the first until the end of the second iteration, there was a slight decrease in average work motivation, but significant difference between the districts.

*Person-centredness* is central to the TM, taking the question “What matters to you?” as a point of departure for shared decision making based on recognition of patient values and capabilities. The team members considered themselves competent and committed, and scored quite high on the composite measures of “Prerequisites for person-centredness” and “Care processes” (see Table 7). However, they gave the “Care environment” a lower score, especially the item “supportive organization”, which in practice means trust-based management gets the lowest rating.

**Table 7:** The team member survey: *Part 3a Person-Centred Practice Inventory*

**Subscales (number items)**	**Jan. 2017** **Mean sum score** **(min–max) *N* = 29**		**2018 Jan** **Mean sum score** **(min–max) *N* = 60**	
***1. Prerequisites for person-centredness***				
Professionally competent (3)	4.01^a^ (3.9 – 4.2)	0.63^d^	4.00 (3.9–4.1)	0.42^d^
Developed interpersonal skills (4)	4.17 (3.9- 4.5)	0.68	4.13 (3.9–4.3)	0.63
Being committed to the job (5)	4.25 (4.2—4.4)	0.76	4.02 (3.9–4.2)	0.79
Knowing self (3)	4.15 (4.0–4.3)	0.67	3.95 (3.9–4.0)	0.74
Clarity of beliefs and values (3)	3.60 (3.4–4.0)	0.71	3.62 (3.4–4.0)	0.73
*Sum score prerequisites*	4.10 (3.4–4.5)	0.84	3.97 (3.4–4.3)	0.86
***2. Care processes***				
Shared decision-making (3)	4,05 (4.0–4.2)	0.75	3.95 (3.9–4.0)	0.59
Engagement (3)	4.17 (4.0–4.3)	0.74	4.0 (3.8–4.2)	0.62
Having sympathetic presence (3)	4.19 (4.1–4.4)	0.66	4.1 (4.0–4.1)	0.71
Providing holistic care (3)	4.20 (4.1–4.3)	0.82	4.03 (3.9–4.3)	0.88
*Sum score care processes*			4.03 (3.8–4.3)	0.86
***3. Care environment***				
Skill-mix (3)	4.01 (3.8–4.4)	0.48	3.93 (3.8–4.1)	0.60
Shared decision-making systems (4)	3.89 (3.6–4.3)	0.63	3.64 (3.3–4.0)	0.78
Effective staff relationships (3)	4.10 (4.0–4.3)	0.85	3.92 (3.9–4.0)	0.79
Power sharing (4)	4.00 (3.6–4.0)	0.78	3.58 (3.3–3.9)^b^	0.85
Potential for innovation and risk taking (3)	3.83 (3.7–4.0)	0.59	3.54 (3.3–3.8)^b^	0.48
The physical environment (3)	3.83 (3.5–4.2)	0.77	3.73 (3.5–4.1)	0.63
Supportive organisational systems (5)	3.50 (3.2–3.9)	0.75	3.18 (2.8–3.4)	0.84
Working with patients´ beliefs and values (4)	4.05 (3.8–4.2)	0.81	3.96 (3.8–4.1)	0.76
*Sum score care environment*	3.89 (3.2–4.3)	0.90	3.66 (2.8–4.1)^c^	0.91

^a^Scale scores: 1 = “totally disagree” to 5 = “totally agree”

^b^Statistically significant difference between first and second iteration

^c^Statistically significant difference first and second iteration (*p* = 0.3, Cohens D = -0.53; i.e. lower score second iteration)

^d^Chronbachs alpha subscales

#### Determinants of innovation (MIDI)

##### Socio-political context: regulations and legislation:

When asked if the TM fits well with current laws and regulations, 64.4% answered agree or completely agree, while 36.6% answered disagree or neither agree nor disagree. This corresponds to findings mentioned above, indicating uncertainty among many team members regarding the TM itself and its legal and administrative aspects, and thus also failure in systematic preparation of the teams.

##### Factors associated with innovation (TM):

Team members in both iterations believed that the TM was relevant to their patients, identified as a facilitator, and that the model is professionally sound and knowledge based. They also found to a varying extent that the trust model has an acceptable level of complexity, that it fits well with how they usually work and that it produces visible results. Factor 3, completeness of the model, was identified as a barrier, indicating that the organization is not well prepared for working according to the TM (Table 8).

**Table 8:** The team member survey part 4b: Factors associated with the Trust Model

**Factor**	**Item**	**TD/D (%)**	**A/TA (%)**	**Mean (SD)** ***N* = 60**	**Range**^a^	**Median**
1. Procedural clarity	The TM is described in clear steps / procedures	11.7	63.3	3.63 (0.9)	1–5	4
2. Correctness	The TM is based on factually correct knowledge	3.0	71.6	3,86 (0.8)	2–5	4
3. Completeness	The TM provides all the information and materials needed to work with it properly	^c^**40.0**	25.0	2,70 (1.1)	1–5	3
4. Complexity	The TM is too complex for me (reversed scale)^b^	11.7	58.4	3.53 (0.9)	2–5	3
5. Compatibility	The TM is a good match for how I am used to working	18.3	53.3	3.41 (1.0)	1–5	4
6. Observability	The outcomes of the TM are clearly observable	18.3	46.7	3.36 (1.0)	1–5	3
7. User relevance	I think the innovation is relevant for the service users	1.7	**80.0**	4.00 (0.7)	2–5	4

*TD/D* Totally disagree/disagree (barriers), *A/TA* Agree/totally agree (facilitators)

^a^Scale from 1 = totally disagree to 5 = totally agree

^b^Reversed—high score means not complex

^c^In Bold—reach the threshold to be a facilitator or barrier

##### Factors associated with team members, patients, and relatives:

The team members were asked about how the TM works for them and how they think it works for patients and their relatives (Table 9). They considered it particularly important that the TM makes it possible to provide services according to the patients’ needs, which they believe is the case. The outcome expectations were identified as specific facilitators. They also found that the TM complies with their responsibility as professionals, that they use their professional skills better and that the work became more exciting. They did not see major disadvantages of the TM. They also believed that they could count on support from colleagues to a fair extent.

**Table 9:** The team member survey part 4c: Factors associated with team members, patients and relatives

**Factor**	**Questions**	**TD/D (%)**	**A/TA (%)**	**Mean (SD)** ***N* = 60**	**Range** ^a^	**Median**
Benefits/drawbacks	In the following we will ask you to what extent the Trust Model does have personal benefits/drawbacks for you?					
8a. Personal benefits	Mostly benefits	8.3	58.3	3.55 (0.9)	1–5	4
	I make better use of my professional competence	8.4	68.6	3.83 (0.8)	1–5	4
	The TM makes my work more exciting	5.0	70.0	3.76 (0.9)	1–5	4
8b. Personal drawbacks	Mostly drawbacks (reversed scale; high mean score implies few drawbacks)	13.3	55.1	3.39 (1.1)	1–5	2
	It makes my job more demanding (reversed scale; high score means not very demanding)	13.3	66.7	2.28 (1.0)	1–5	4
9. Outcome expectations	a. It is important the TM provides services according to the users’ needs	6.7	**93.4**^b^	4.43 (0.7)	2–5	5
	b. I expect that the TM provides services according to the users’ needs	6.7	**91.7**	4.33 (0.7)	2–5	4
10. Professional obligation	10. Arbeidet i Tillitsmodellen er i tråd med mitt ansvar som sykepleier / helsefagarbeider / ergoterapeut / fysioterapeut / annet helsepersonell (sett strek under din kategori) My work in the TM is in line with my professional obligations	15.0	76.7	4.00 (0.7)	2–5	4
11. User satisfaction	a. The users will generally be satisfied if I work according to the TM	1.7	66.7	3.86 (0.7)	2–5	4
	b. The users’ relatives will generally be satisfied if I work according to the TM		71.7	3.93 (0.7)	3–5	4
12. User cooperation	a. The users will generally cooperate if I work according to the TM	5.0	63.3	3.72 (0.8)	2–5	4
	b. The users’ family will generally cooperate if I work according to the TM	3.3	58.3	3.68 (0.7)	2–5	4
13. Social support	I can count on adequate assistance from my colleagues if I need it	10.0	58.3	3.67 (1.0)	1–5	4
	**Sum score (Cronbach´s alpha = 0.79)**			**44.00 (5.5)**	**29–59**	

*TD/D* Totally disagree/disagree (barriers), *A/TA* Agree/totally agree (facilitator)

^a^Scale from 1 = totally disagree to 5 = totally agree

^b^In Bold—reach the threshold to be a facilitator or barrier

##### Factors associated with the organization:

Half of the team members found that they had too little background knowledge to work according to the TM when they started. Asked whether management had formalized the TM, 37 answered “yes” (67%), 20 “do not know” (33%) and 3 “no” (5%). Asked if there were other change processes going on at the workplace during the implementation of the TM, such as reorganization, mergers, cost cutting, or other innovations, 40 informants answered “yes” (77%), 12 “no” (23%) while 8 did not responded to this question. This indicates that there was considerable uncertainty among many team members about the TM as a management model, and that other organizational change processes were happening to a relatively large extent parallel to the TM iterations, which usually implies relatively poor conditions for innovation and implementation. Several factors were identified as barriers: Replacement when staff leave, staff capacity, financial resources, time available, material resources and performance feedback (see Table 10). This suggests that team members found the organization was not sufficiently prepared for working according to the TM intentions.

**Table 10:** The team member survey part 4d: Factors associated with the organization

**Factors**	**Questions**	**TD/D**	**A/TA**	**Mean (SD)** ***N* = 60**	**Range**^b^	**Median**
20. Replacement when staff leave	In my organization, there are arrangements in place so that TM staff who leave the organization are replaced in good time by employees who are/will be adequately prepared to take over	**47.3**	20.0	2.62 (1.1)	1–5	3
21. Staff capacity	There are enough people in our organisation to work according to the TM as intended	**49.1**	20.3	2.41 (1.1)	1–5	3
22. Financial resources	There are enough financial resources available to work according to the TM as intended	**40.6**	10.2	2.37 (1.0)	1–5	3
23. Time available	Our organisation provides me with enough time to include the TM as intended in my day-to-day work	**55.9**	18.7	2.48 (1.1)	1–5	2
24. Material resources and facilities	Our organization provides me with enough materials and other resources or facilities necessary for working according to the TM as intended	**30.6**	32.2	2.88 (1.1)	1–5	3
27. Information on how the TM works	It is easy for me to find information in my organization about the TM	8.4	73.3	3.70 (0.9)	1–5	4
28. Performance feedback	In my organisation, feedback is regularly provided about the progress of implementing the TM	**20.3**	49.2	3.32 (1.2)	1–5	3
	**Sum score (Cronbach's alpha = 0.84)**			**29.94 (5.2)**	**9 -34**	

*TD/D* Totally disagree/disagree (barriers), *A/TA* Agree/totally agree (facilitator)

^a^In bold—reach the threshold to be a facilitator or barrier

^b^Scale from 1 = totally disagree to 5 = totally agree

### Team-members experiences and perspectives – qualitative exploration

The summarized findings are presented from the three IGP process workshops during the second iteration, where all four districts were represented with members of all eight teams at each workshop.

#### Workshop 1 – Sharing experiences

The action research purpose of the first workshop (October 2017) was to share experiences across teams and districts, and to learn from each other's ideas and experiences of factors facilitating or preventing the innovation process, trust, and service quality. Forty-nine team members from the four districts participated.

##### Value alignment:

Table 11 displays how team members at the end of the second iteration perceived the alignment in practice with the TM principles and values as developed by the preceding bottom-up process and decided on by the steering group. These principles and values should ideally be implemented by all teams, i.e., that the answer to the first 13 questions should be “yes”. We see that this is not the case. We also see that relatively few report that they have re-evaluated patient needs and involved patients more than before, which might have been expected at this point. In response to open questions and plenary experience sharing, the team members reported that what worked well both for them and for the patients was the multidisciplinary co-operation in teams, which was experienced as inspiring and motivating. However, several teams reported insufficient competence and minimum staffing, which did not work well neither for the teams, being dependent on substitute staffing, nor for the patients, wanting predictability and continuity.

**Table 11:** Experienced alignment with TM principles and values as reported in team member workshop^a^

Statements based on the defined TM principles (1–13) and the supposed innovation support and effects (14–19)		yes	no	don’t know
1	My team is responsible for all users in our area	28	13	2
2	My team include both executive officer, occupational therapist and physiotherapist as permanent members	38	5	
3	Practical assistance is integrated into the team	22	20	1
4	All permanent members of my team report to the same leader	35	5	2
5	The authority to make decisions in my team is by the team leader	18	19	6
6	We are about to implement new care decision plans for each single user, starting with «What is important to you?»	17	22	3
7	The team receives additional (special) competence and supervision when needed	27	6	10
8	The team make decisions on homecare service provision	20	21	2
9	The team makes home care plans and follow up procedures	27	10	4
10	The team makes adjustments of the users service needs (in cross-disciplinary meetings)	41	1	-
11	The team is responsible for creating the staff rosters	5	35	3
12	Team members receive supervision and advice within the team	22	13	7
13	The team follow up users and keep contact with hospitals and other partner institutions	20	12	10
14	The team has sufficient technology support	13	15	13
15	We have re-evaluated service needs together with all users	3	37	3
16	In my team the cross-professional co-operation works well	36	3	4
17	I feel that myself and the team have good leadership support	13	24	4
18	I feel that the team as a whole has the necessary competence to give holistic care	28	9	6
19	I feel we involve the users more than before	9	27	7

^a^*N* = 49. Members of all teams in the four city districs took part in the workshop (Nov. 2018)

##### Leadership support:

All teams agreed that team leader support was essential to functioning well as a self-managed cross-disciplinary team. Some teams reported having leaders present facilitating cross-disciplinary communication and service decision making. Other teams reported lack of such support; that the team leader often was absent, that standards for team decision making were unclear, and that they felt they were given the responsibility of making care decisions and developing staff rosters without being trained or prepared for it. Two teams also reported that essential administrative competence was withdrawn from the team during the second iteration, which made work more difficult and at times chaotic.

##### Team leader openness:

Another issue of trust was the degree to which the team leader was open to quality concerns and change proposals, for instance concerning team competence, staffing, procedures, patient safety or quality of care. A team leader who was present, encouraging, appreciated open discussion and team decision making was reported to be an important TM facilitator. Conversely, the TM worked less well for the teams when the leader considered team members’ concerns and proposals for improvement as criticism, and the teams were left alone to find solutions to questions of patient safety or quality of care. This was especially so when the problems were perceived to be of a systemic nature, like lack of clear care decision procedures, sufficient staffing, or a well-functioning patient record system.

##### Team members’ advice to management:

The participants responded individually to a question as to what advice they would give to management to make the TM work even better for them and for the patients. The most frequent answers were ensuring a) sufficient staffing, b) team-leader being present and participating in the daily work, and c) team competence building when needed. In addition, the needs for d) clarification of tasks and procedures, and e) sufficient time for administration were frequently raised.

#### Workshop 2—Trust, motivation, and cooperation

At the second workshop (November 2017) the purpose was to clarify the team members roles in the team and how to work together across competencies to enhance the patients' feelings of security, self-determination and coping. All eight teams were represented with 43 team members in all. The individual questionnaire focused on trust and co-operation in teams. In the plenary discussion trust was reported to be a key motivating factor facilitating cooperation in teams, provision of quality care and the TM innovation process in general. Trust was related to relying on others at different levels, the team leaders relying on the teams, the team members relying on colleagues, and the patients relying on the team members and the service. Emphasis was placed on honesty, openness, communication, searching for shared solutions. Trust was also perceived as having faith that everyone is doing their job and doing their best. It seemed to be a general experience across the teams that having less patients to visit made it possible to establish a more trustworthy relationship with each person.

A surprising discovery at this workshop was that not all teams worked according to the new, person-centred procedure, although this was central to the TM and that a new template, based on the “What matters to you?”-question and the principle of shared decision making, was developed during the first iteration. Another surprising discovery across teams was that the new procedure was not supported by the digital patient record system, which was considered a major barrier to TM practice. The teams also reported a lack of a digital communication tool, making cross-disciplinary communication within the teams and shared decision at the patients home possible. This was considered to make changing practice unnecessarily difficult and decelerated the implementation of the TM.

#### Workshop 3: Roles and communication

The third and final workshop of the second iteration (January 2018) had 35 participants from three districts. Taking the results from the previous workshop as a starting point, the theme was the initial interviews with new patients. Key points were how to create trust and good communication in the interview situation, identify what is important for the patient, and create a shared decision-making process. It was reported that following this procedure and showing interest in what was important to the patients, seemed to improve the relationships and had a motivating effect on patients' potential for self-management, working more actively to improve their health condition. Some also reported radical positive results after some time, like long time patients no longer needing homecare. Others suggested the need for competence building and skills training concerning shared decision-making, especially for new team members. They also reported that sharing experiences across teams and learning from others’ experiences was inspirational and useful.

### The managerial perspective

Individual interviews were conducted with ten managers half-way into the second iteration, in December 2017. Problems concerning organizational change were directly or indirectly core issues in most of the interviews. The perspectives among the managers were quite diverse. They all identified complexity as a central issue, that abolishing the PPS and establishing and formalizing the TM represented a giant organizational step. Most of the leaders pointed out that the two iterations had proven that the transition from PPS to trust-based management and delivery was far more complex than expected. Ten different organizational issues challenging the innovation process were identified: (1) *organizational complexity,* (2) *organizational agent autonomy*, (3) *competing organizational logics*, (4) *balancing trust and control*, (5) *leadership anxiety*, (6) *parallel change projects*, (7) *team member mindset*, (8) *team member competence*, (9) *team capacity* and (10) *digital support*. An overview of these challenges as reported by the managers is presented in Table 12.

**Table 12:** Overview of organizational issues challenging the innovation process as reported by managers

Managerial issues	Subthemes and illustrative quotes
*1. Organizational complexity*	• Piloting the TM through two iterations across four autonomous districts was much more complex than expected
*2. Organizational autonomy*	• The feeling of ownership among leaders on different levels varied between districts, influencing local choices during the two iterations
*3. Competing organizational logics*	• The TM challenge the existing NPM principles of management by objectives, but without really replacing it • *“We get conflicting steering signals from above”*
*4. Parallell change projects*	• One's own and other districts were involved in parallel organizational change processes while running the TM iterations, which made it hard to give the necessary priority to the TM • “*There are so many changes happening at the moment*”
*5. Balancing trust and controle*	• Challenging finding the balance between trust and control, giving rise to considerable uncertainty concerning trust-based management • “*As a leader you cannot just say, ‘I trust you with the full responsibility’. You need to govern as well*.”
*6. Leadership anxiety*	• Feeling of unease or uncertainty concerning the ambitions of the TM project and their new role, • A fear that trust-based management would jeopardize budgets and/or service quality. “*It was so much anxiety*.”
*7. Team member mindset*	• Need for a change of staff mindset or culture • Team members were used to performing detailed tasks defined by others • Team members are not used to exercise their power of discretion, co-operate closely across professions and make shared decisions with patients in a team setting. “*This is a huge cultural change, and it will take time*”
*8. Team member competence*	• Hard to trust their teams with the intended full responsibility for making decisions and providing the daily care • Young RNs lack educational preparation for working in self-managed teams • A need for team development and competence building in areas like multi-professional co-operation, problem solving, person-centred communication and shared decision-making
*9. Team capacity*	• Concerns about the staff capacity. “When sick leave occurs it becomes chaos because it is so vulnerable.” • A fear of pressure from above to “realize benefits” by reducing the staff to a minimum as soon as the TM was implemented
*10. Digital support*	• Patient record system did not support the TM and the new way of working • “*It [the patient record system] is too cumbersome, I mean, it is too much a set up for the PPS*.”

#### Leadership anxiety

One of the top managers, having observed the process from the very start, highlighted the leadership anxiety when the project was launched, especially among top managers and union leaders: “*It is a very demanding project. And it was very much uncertainty in the beginning about what should come out of it, and it was quite much …, eh, yes, it was much anxiety*”. According to this manager, the leadership anxiety was widespread, the TM challenging managers’ roles, responsibilities, and control over decision-making processes. In addition, the level of political ambitions made top managers uncertain: “*It was a project with very high ambitions, they [the politicians] went very high, very fast and high, and that lead to both a high-level anxiety and a high level of unease.*” They also pointed at the organizational complexity as a source of uncertainty and anxiety: “*It is a very complex organization (…). This caused considerable concern on behalf of the team members’ unions, and with four chains of command [four city districts] or five or six or very many, it’s quite natural that it gives rise to such anxiety*”. On the other hand, having experience with running large projects in other organizations, the same manager stressed that organizational anxiety was a recognizable feature of change processes: “*This is nothing special for the Oslo municipality. Not at all.”*

#### Leadership trust in the TM

At the same time, all the managers said they found the TM promising. When being explicitly asked, they declared that they preferred the TM to the PPS, and no one wanted to return to the PPS. They gave a series of reasons for this. Multiprofessional competence in the teams enhanced the quality of care and of care decisions. Co-operation across professions was motivating for the team members. Having both executive officers, occupational therapists and physiotherapists as permanent members of the team seemed to be a preferred solution, perhaps dividing their time between two teams.

#### Roles and responsibilities

A challenge, according to the managers, was finding the balance and distribution of responsibilities. The teams tried out different models, some with collaborative decision-making, some with clear distribution of individual roles and responsibilities, and some teams somewhere in between.

Several leaders emphasized that starting with “what matters to you?” represented a new mindset both for team leaders and team members. A barrier throughout the innovation process agreed upon was that the teams were not sufficiently prepared for the new practice of self-managed team co-operation, care decision-making and person-centred practice.

Some managers reported that even registered nurses seemed to find it difficult to take on a professional responsibility and exercise discretion in questions of care delivery and distribution. One of the top managers raised the question if nurses’ professional judgment and autonomy was *unlearned* after many years in the provider role. Another pointed to the fact that the written TM procedures for allocation and distribution of services were still in the making (at the end of the second iteration), and that this lack of clear guidelines through both iterations might have caused considerable confusion in the teams.

In addition to lack of preparation, training, and clear procedures, also the digital patient record system was reported to represent a barrier, originally developed within the PPS framework and unaligned with the TM principles of team autonomy, person-centredness and shared decision-making. Even though this was recognized by the steering group from the very beginning, no alignment efforts were made throughout the two iterations. We were unable to unveil any explanation for this, except for some managers’ references to the complexity of the municipal organization and the tension and unclear distribution of responsibilities at the system level, between the municipal top management and the relative autonomy of the city districts.

#### The team-leader

The team-leader role was a key topic in most of the interviews. It was a general view that a barrier to the innovation process was that the team-leaders were not trained for their new role. On the other hand, in practice, three different approaches or roles were tried out during the two iterations, a) a distant, administrative role, b) a coaching role outside the team, or c) a coaching role inside the team. The informants had different views on this issue. Some expressed disappointment concerning teams wanting their team-leader to be regularly present at meetings and taking an active part in decision-making processes They found “lack of courage” among team members to act according to the intention of self-management to be a barrier to the development of the service innovation. Others found the teams’ need for a present and participative team-leader quite natural given the teams’ full responsibility for care decisions and delivery, and saw the coaching leader inside the team as a key facilitator. This last view is congruent with the results from the workshop process interviews. One team-leader, having chosen this coaching team-leader role inside the team, explained how she had changed her way of thinking as a leader during her preparations for the second iteration:

*I have turned completely around. I am now a ... part of the team. It is not clearly me and them. Now I sit in another place in the group. I don’t conduct the morning reports anymore. I don´t conduct the user meetings. Those are conducted by the team. We have had a lot of team meetings on how we want our team to be. (…) And I have taken on a more passive role, letting the others come forward, more ... professional discussions, reflections, searching for solutions. The chairperson role changes continuously ...*

### Making sense of the findings and constructing the innovation framework

We have presented the results of the analysis of the multitude of data sources concerning what matters the most to the patients (“patient values”) and facilitators and barriers to trust-based service innovation as experienced by participating team members, team leaders and managers in different roles and on different levels in the municipal organization. The main result is that trust-based innovation of homecare is a complex and multileveled organizational operation, involving a series of facilitators and barriers on four levels – patients, team members, team leaders, and service & system management. These findings are synthesized and made sense of through the construction of an integrative four-level framework for trust-based service innovation of homecare (Fig. 1).

## Discussion

To our knowledge the present study is the first one exploring the complexity of trust-based organizational change in homecare and developing an organizational framework for trust-based service innovation in municipalities. We discuss the findings at each of the four levels of the framework drawing on empirical research and theories of person-centredness, motivation and organizational complexity.

### Patient values

At the centre of the proposed framework we find the patient values, i.e., the key findings of the patient survey. The patients value a) predictability and continuity, b) service providers with competence and skills, and c) that the staff are friendly and helpful. These are the prerequisites that according to the patients need to be fulfilled for them to have trust in and be satisfied with the service, which was the declared aims of the innovation. Patient values are placed at the centre of the figure, consistent with frameworks for person-centred healthcare practice [21, 47] and leadership [48, 49].

### Team members prerequisites

The 1^st^ circle around the patient values displays the results concerning team prerequisites, i.e., key qualities facilitating patient trust and the TM innovation. Through analysis of the patient and team members’ surveys and the qualitative explorations of the team members’ experiences *eight such key qualities were* identified. Five of these were coined as combinations of individual qualities (reliable, competent, familiar, caring, and person-centred) and characteristic concrete behaviours (like to arrive according to schedule, know what to do, be attached and helpful, and make shared decisions). The remaining three were coined as combinations of team qualities (co-operative, autonomous and sustainable) and concrete and dynamic team processes (cross-disciplinarity co-operation, team decision-making and quality assurance). Some of the individual qualities overlap to a certain extent, like being competent, caring, and person-centred. The reason for making these distinctions is to highlight aspects of professional qualities pointed to by patients and team members as the most important separate facilitators of trust (and as barriers, when not present). These findings are in line with the political ambitions of the TM, empirical homecare research [50, 51] and prerequisites of person-centred practice [21].

### Team leader prerequisites

The 2^nd^ circle displays the findings concerning team leader prerequisites, i.e., key qualities and behaviours that facilitate team leader engagement with other team leaders and with team members. These are the condensed results of the analyses of the team members survey, the team workshops, participatory observation notes and leadership interviews. Five such prerequisites were identified. We termed the main facilitator “Participative team leadership”, the adjective “participative” indicating the function of being present and participate in the team meetings, prioritizing dialogue, and facilitating multidisciplinary team member participation and decision-making. These findings are comparable both with the literature on multidisciplinary teams [52] and on management and organizational risk, pinpointing that well-functioning high risk organizations (which a homecare service might be considered to be) are characterized by leaders creating a culture where questioning is ok and people feel free to raise concerns [53]. However, a recent Swedish study on trust-based management in homecare found that the quality assurance task was performed by frontline team members, while the first line management was characterized by checks instead of controls [54]. This seems to be the preferred position also of some of the team leaders in our study, supported by some managers considering the teams’ reported need for a present team-leader as a sign of weakness or lack of courage to take responsibility. However, the assumption that individual team-members will take responsibility when decisions have to be made is not supported by the literature [55]. In our study, we found the practice of some of the team-leaders of leaving quality assurance and other decision-making tasks to the team members, to be a barrier rather than a facilitator of the innovation process. The team members’ experienced need for a present, participative team leader is also in line with recent research on the transformation to self-managed teams in healthcare, indicating that the team-leader role progresses through different phases, from a more active role in the beginning to a more coaching role over time [56]. This need for a participative team leader might be explained by Self-Determination Theory (SDT), postulating experienced competence, relatedness, and autonomy to be key intrinsic motivational factors, and when these factors are thwarted results in diminished motivation [57, 58]. SDT may also shed light on the four additional identified team leader prerequisites (team communication, role clarification, decision making, staffing, quality assurance), including relational, autonomy supportive and competence building elements. These findings are consistent both with the results from a recent meta-analysis on work motivation, concluding that in order to achieve team members motivation and performance, organizations “should nurture autonomy support from colleagues or supervisors” [59]. It is also consistent with the results from a recent empirical hospital-based study, revealing a complex pattern of links between leadership autonomy support and innovative team members’ behaviours [60].

### System and management prerequisites

The 3^rd^ circle displays the system and management prerequisites, i.e., system properties facilitating the TM innovation process when present (and thwarting it when not). These are the results the analyses of the leadership and team workshop interviews and participatory observation notes. Six such prerequisites were identified.

The main prerequisite on the system level was termed “trust-based organizing and management”. There are many ways to understand the emerging concept of trust-based management [54]. By “trust-based” we here mean the primacy of the value of trust both as an *organizing principle* (self-managed teams), a *leadership style* (trusting employees’ with decision-making powers) and as a *management objective* (to increase citizen trust in the home-care services). The reason for emphasizing trust-based management and organizing as a key facilitator on the system level was the reported competing institutional logics between the established PPS principles of management by objectives and TM innovation principles. This hybrid organizational approach and lack of coherence concerning management principles, were perceived as a barrier to the innovation both by managers, team leaders and team members.

The most surprising finding on the system level was the reported widespread anxiety among managers. The relationship between leadership anxiety and trust is a complex one, and strategies for coping with anxiety in organizations depends on multiple factors [61]. According to the managers themselves, the organizational complexity of the municipality and the many different agents involved were important reasons for their anxiety and fear of losing control. This is paradoxical. According to Luhmann [28] trust is a mechanism for *reduction* of social complexity. According to recent research on organizational culture [62], anxiety in organization might be caused by factors like result-orientation under time pressure, lack of consensus about norms and mismatch between organizational norms and values. In our case, the managers were facing all these three challenges, to a high degree triggered by the organizational hybridity. However, struggle between competing institutional logics is a well-known issue in healthcare organizations [63].

Our study clearly shows that a political decision from the top of making trust the primary organizing principle is not sufficient to secure trust-based innovation on lower levels of the organization. This finding is consistent with the Copenhagen trust reform study [7, 25]. The five additional prerequisites at the system level (sufficient staff resources, aligned patient record system and other digital solutions, competence building resources and quality assurance across teams and city districts) represent concrete system elements reported to facilitate the TM innovation (and thwarting it when not present). The importance of careful planning, systemic support and clear decision-making procedures is in line with research on determinants of innovation [36, 37], research on how to build trust and cooperative relations in decision-making teams [64], and also with research on service innovation in other areas of municipal healthcare [65, 66].

### The organizational trust perspective

The overall objective of this study was to develop a comprehensive model of trust-based service innovation of municipal homecare. In the following we will discuss the results and proposed framework in the light of the integrative model of organizational trust [11, 29], postulating *ability*, *benevolence* and *integrity* as the three essential qualities determining the degree of trust in an organization, here the homecare service. These three characteristics are also found as facilitators in our study.

*Ability* might mean something slightly different at the different levels of the proposed framework: On the team level it is an enabler of competent and person-centred care; on the team-leader level it gives rise to the necessary support, and on the management level ability can lead to the provision of the necessary system resources.

Also the virtue of *benevolence* might mean something slightly different on different levels; On the team level it relates to care for the individual patient, but also to the approach to co-operation with colleagues; on the team-leader level it represents being present, showing interest and helping each team member to succeed, and on the management level to have the patients’ best interest in mind by supporting their staff and providing the necessary systemic support, which to some might imply putting personal anxiety aside and making brave decisions in support of the innovation.

*Integrity*, on the other hand, might primarily mean one thing, namely “walking the talk”, i.e., adhering consistently to the value of trust as an organizing and managerial principle. On the team level this might imply listening to the patients’ needs, providing person-centred care and making shared decisions; on the team-leader level it relates to clarifying procedures and giving consistent support for autonomy, and on the management level it ensures system support of the innovation and seeking trust-based solutions to competing organizational logics and values when necessary. Our study indicates that organizational integrity might be the main challenge to successful trust-based service innovation in homecare.

### The complexity of healthcare innovation

Studying this complexity and the mechanism of trust in the municipal healthcare context has been challenging. There were few models to learn from. Even though the complexity of healthcare management and practice has been recognized since the beginning of this century [67], and complexity theory at that time had entered the field of organization studies [68–71], there are few empirical studies and a lack of theorizing and method development in the field of healthcare [72].

This study has evoked the question of how to describe the complexity of a healthcare organization, the organizational “chaosmos”, as it is termed by Tsoukas [73]. There were significant tensions between the ethical *intentionality* of the TM service innovation (e.g. trust, person-centredness, quality of care), the organizational *system* (e.g. digital solutions, quality procedures, patient record system) and the organizational *culture* (e.g. competing organizational logics, diverse leadership approaches) during the two iterations. We observed the emergence of positive emotions (like interest, joy and enthusiasm), negative emotions (like uncertainty, anxiety and anger) and social interaction elements (like role conflicts, power play, resistance, rivalry, mutual support and professional co-operation) within and across the eight teams and their respective management surroundings. These emotions influenced the development of the agents’ perception of trust and the TM innovation process. It would be far beyond the scope and resources of this study to identify and analyse in depth the complexity of these fluid and dynamic processes.

Describing complexity necessarily implies simplification. Here we have chosen *person-centredness* as the basic principle for organizing the findings (Fig. 1), in accordance with the basic ethical *intentionality* of healthcare organizations, i.e., providing care for those in need and taking what matters to the individual patient as the starting point for doing so. Having systematized the findings and developed the framework, the organizational complexity might seem less complex. We therefore need to add that this framework is limited to key prerequisites that – according to our findings – facilitate trust-based innovation of homecare practice when present (and thwart it when not).

### Strengths and weaknesses of the study

The complexity of organizational innovation in healthcare goes deep and may seem beyond discursive analysis and description. Possible strengths of this study are the recognition of this complexity, the longitudinal mixed methods design, and the multiprofessional composition of the research team, including scholars with backgrounds in nursing, psychology, sociology, innovation, literature, leadership, ethics and organization studies, trying to keep it all together. A weakness of the study is that the complexity of the subject matter goes deeper than we have been able to analyse and display. In fact, most of the elements identified and represented on the different levels of the proposed framework, like patient values, team reliability and competence, the team leadership role and the different system and management prerequisites, might have been made the focus of separate in-depth studies.

## Conclusions and implications

Organizing homecare services in self-managed multidisciplinary teams according to the value of trust seems like a feasible alternative to the PPS. The proposed framework is designed to improve our understanding of critical prerequisites that may affect trust-based service innovation processes and quality of the results, and can serve as a tool when planning trust-based innovation strategies. It may also be used as a checklist for successful implementation and iterative improvement of trust-based organizing and management processes in homecare. Further empirical research is needed to explore the usefulness and validity of the framework and its possible applicability to trust-based and person-centred service innovation in other areas of healthcare. The importance of iterative involvement of affected parties should not be underestimated. Complex, value-based organizational innovation and change (like the TM) challenges existing institutional logics (like NPM) and the organization’s ability to cope with institutional hybridization. The complexity of organizational innovation in homecare is understudied. The importance of recognizing the complexity of trust-building and the inherent slowness of radical service innovation should be further explored.

## Data Availability

The quantitative data sets, interview guide and questionnaires can be made available by corresponding author on reasonable request. The qualitative data consist of transcribed interviews and field notes in settings with a limited number of participants, and can for privacy reasons not be made available.

## Abbreviations

BMcC: Brendan McCormack (author)
ERN: Etty R. Nilsen (author)
HE: Hilde Eide (author)
JHD: Janne H. Dugstad (author)
MIDI: Measuring Instrument for Determinants of Innovations
MKG: Monika K. Gullslett (author)
NPM: New Public Management
PPS: Purchaser-provider split
SDT: Self-determination theory
TE: Tom Eide (author)
TM: Trust Model
